# Data losses and synchronization according to delay in PLC-based industrial automation systems

**DOI:** 10.1016/j.heliyon.2024.e37560

**Published:** 2024-09-06

**Authors:** Ayah Hijazi, Mátyás Andó, Zoltán Pödör

**Affiliations:** Institute of Computer Science, Faculty of Informatics, ELTE Eötvös Loránd University, Budapest, Hungary

**Keywords:** PLC, Node-RED, Synchronization, Time delay, Missing values

## Abstract

PLC (Programmable Logic Controllers) based data collection is integral to industrial automation and data acquisition processes. The pipeline – between the device and the database – is a complex system with many components and often causes some time delay, due to the synchronization and the applied hardware and software components. This delay can also lead to data loss on the database site. In this study, we aimed to examine this problem connected to the synchronized behavior of four counter networks within the TIA software for PLCs, particularly focusing on the implications of a 1 Hz (Hz) clock frequency on counter synchronization. Meticulous experiments were conducted and the integration of Node-RED, as an instrumental tool in industrial automation, facilitated counter-behavior monitoring. Recorded values of counter and timestamps were meticulously stored in four separate databases (MSSQL, MySQL, MongoDB, and Apache Cassandra) for comprehensive data analysis. Throughout the experiments, inconsistencies in the counter values were encountered, leading to the discovery of missing values that varied across the ten tests. To detect the reason, a unique delay calculation method was developed. According to our results, we were able to do post-synchronization with millisecond-level accuracy. It can help reveal the missing values that depend on the Node-RED and the PLC cycle time differences.

## Highlights

• Systematic data analysis in the case of SQL and NoSQL communication with PLC.
• Created a predictive model for anticipating missing values during data transfer from PLC based on the time differences.
• Introduce the possible compensation values – as a key factor – to determine the delays.
• Create a unique method that can determine the un-synchronization between PLC and database according to the collected data.
• Provided valuable insights for data transmission and synchronization in industry, to enhance efficiency and decision-making.

## 1 Introduction

Industrial data processing and gathering have long faced several difficulties that have endured throughout time, like building a stable connection between the database and the data source, handling and storing the huge amount of data without any loss, managing various data formats, maintaining data quality, assuring security, and attending to real-time processing requirements [1]. While some of these difficulties have been temporarily resolved, the constantly shifting industrial environment keeps reintroducing issues.

In this research, we focus on addressing these challenges by building a complex pipeline between Programmable Logic Controller (PLCs), Node-RED, along with four different databases (MSSQL, MYSQL, MongoDB, and Apache Cassandra). PLCs are integral in industrial automation, serving as the backbone for data collection from various sensors and machinery. Node-RED, an open-source flow-based development tool, facilitates the integration of PLCs with databases, enabling the visualization, processing, and storage of industrial data.

However, we encountered specific issues during this integration, such as missing values in the datasets. These missing values can arise from various sources, including sensor malfunctions, data entry errors, or transmission failures, and can lead to incomplete analyses, biased models, and incorrect predictions, significantly affecting decision-making processes. Additionally, there were delays in data synchronization between the PLCs, Node-RED, and the databases, which exacerbated these issues, causing inconsistencies and misalignment of data. These delays might be due to network latency, processing bottlenecks, or system overloads, further complicating real-time processing and decision-making.

Overall, our work aims to address these challenges by leveraging PLCs, Node-RED, and careful selection of databases to ensure data integrity, reliability, and efficiency in industrial data processing systems.

### 1.1 PLC – communications – database as the technical background

PLCs are integral to industrial automation, designed for data collection with the aims of cost reduction and product quality improvement, while also being adaptable to hazardous environments and presenting user-friendly programming [2]. PLCs serve as the backbone of control systems and industrial automation, permitting real-time control and supervision to optimize production processes. Originating during the Industry 3.0 revolution in the late 1960s, PLCs remain a vital technology, especially in conjunction with robots in modern factories [3].

After PLC data collection, it is crucial to transmit it to a well-prepared database via an appropriate communication channel. One common choice is Ethernet/IP, an industrial communication protocol based on Ethernet technology, recognized for real-time communication, developed by Rockwell Automation in 2001, with a data transfer rate of 100/10 Mbit/s. Ethernet/IP utilizes the Common Industrial Protocol (CIP) for various applications, including programming, diagnostics, data collection, PLC data exchange, and I/O communications [4,5].

CIP has two types of connections: Explicit CIP and Implicit CIP. Explicit CIP uses TCP/IP for communication and includes unscheduled request/response type messages, while Implicit CIP uses UDP/IP for communication and is scheduled, with an identified Requested Packet Interval (RPI) for data updates [6]. Explicit messaging in CIP provides flexibility but comes at the cost of more complicated setups and event-triggered messages. Implicit messaging is superior for tasks but less flexible as data values are assigned during connection establishment [5].

The assembled data needs a place to be stored so that it can be utilized later or used during the process for different applications. As the huge number of sensors, PLC-s, and other industrial data collectors increase Big Data term has appeared in the industry too [7]. Big Data is usually characterized by the 4 V or 5 V models, which include volume, variety, velocity, veracity, and value [8]. The components and difficulties of successfully organizing and analyzing this enormous amount of data will be covered in this research. The SQL and NoSQL (Not only SQL) based approaches will take into consideration the speed, the noncentralized databases, and the flexibility in processing the enormous quantity of data [9].

In SQL-based databases the scaling is vertical, and the storage of data is stored in tables within a row of individual records. One of the limitations of these databases is that if any kind of data must be modified the whole database should be changed [10]. Relational databases play a leading role in managing transactional, consistent, and well-structured data. Is the biggest advantage and disadvantage of these databases: easy to search, and serve the request quickly, but the structure of the database is fixed, and cannot handle a new sensor [11].

The NoSQL databases are not a single tool or software, but rather a methodology or approach to database management that involves several complementary and competing tools [12]. Moreover, NoSQL embraces the advantages of strong mass storage, a high degree of scalability, dependability, a clear data model, a basic query language, and little support for data consistency. Their primary competitive advantage is the effective management of unstructured data, which traditional relational databases are less equipped to handle, such as documents, emails, multimedia, and social media [13,14].

In the industrial settings of today, we frequently produce enormous volumes of data. Improved data management and analysis techniques must be used to handle and use this data more efficiently. In this article 4 different databases (two SQL-, and two NoSQL-based) are utilized to compare their performance, with a focus on how effectively they can manage increasing input, and various types of data produced by PLC:

• MSSQL Server is an exclusive relational database management system that was created by Microsoft [15,16].
• MySQL is a free and open-source database system. It was created in C and C++, and it comes with MySQL Workbench, a bundled environment that makes it easier to administer MySQL databases and create database structures visually [17,18].
• MongoDB is written in C++ and is document-oriented, the items are stored in sequence as BSON [10,19]. BSON stands for Binary JSON, it supports boolean, float, string, integer, date, and binary types and uses efficient encoding for a variety of data kinds, including numbers [20].
• Apache Cassandra is an open-source distributed database management system constructed to handle considerable amounts of data across multiple servers. Cassandra stores data as a structured key-value store with tunable consistency. Keys can represent multiple values, which are clustered into column families [21].

The decision to include MSSQL, MySQL, MongoDB, and Apache Cassandra is based on the fact that these four databases are often utilized within the top 12 databases for a wide range of applications as shown in Refs. [22,23]. These databases have a variety of features and characteristics that make them suitable for industrial environments [24]. MSSQL and MySQL offer robust features for structured data typical in industrial applications, while MongoDB's schema-less design and Apache Cassandra's distributed architecture provide scalability and fault tolerance. Additionally, MySQL is widely used in web applications, MongoDB in platforms like Google and Facebook, MSSQL Server in enterprise applications, and Cassandra in managing large datasets for companies such as eBay, Netflix, and Instagram, enabling a comprehensive comparison across crucial performance metrics for industrial applications [25]. The purpose of using these two distinct kinds of databases is to evaluate any discrepancies in the data-gathering process and pinpoint any constraints. By analyzing both SQL and NoSQL databases, this study aims to assess their relative strengths and limitations in handling industrial data, thereby providing insights into their suitability for various operational scenarios.

### 1.2 Scientific background

This section delves into the research efforts as it seeks to provide an important response regarding the database most suited for this experiment to identify the root cause of missing values and devise effective solutions to mitigate them. The experiment involves transferring data from a PLC to four different databases to assess their respective benefits and limitations, with a primary focus on data transfer speed and delay occurrence. Choosing the optimal database presents a challenging task, contingent upon the hardware and software infrastructure currently established and closely aligned with the goals of data collection. The industrial goals dictate precise output requirements and the adoption of suitable data processing techniques. Additionally, the constraints and limitations of the selected database platforms are pivotal to these systems, particularly in ensuring speed and reducing latency in data transmission procedures.

In an industrial environment, remote monitoring has become more important due to the lack of skilled employees. PLC communication can extend not only to the Ethernet module but also to GSM. In 2008, the platform utilized the GSM network and a communication protocol that employs SMS messaging to communicate with PLC stations and a Database Server integrated within the system [26] was introduced. Nowadays it is clear that SMS communication is not enough because of the speed and the amount of information. The authors [27] described the advancement of a mobile application for supervising and controlling a 5-axis CNC machine center in real-time, via a cloud-based database. The results of the performance tests showed that Firebase was much faster than MySQL (four times faster) in handling multiple sequential insertions.

Numerous studies have compared the performance of SQL relational databases to NoSQL databases, particularly MongoDB. As MongoDB executes inserts, updates, and simple searches effectively, it is appropriate for dynamic schemas and bigger datasets. Additionally, whereas MySQL is better suited for smaller datasets, MongoDB appears to be successful for insertions and complicated searches in non-relational data retrieval but has disadvantages in development and post-maintenance [9,[28], [29], [30], [31]].

In the study by Van der Veen et al. [32], PostgreSQL (SQL) and MongoDB (NoSQL) were compared for sensor data systems. They found that while PostgreSQL offers better read performance and flexible querying, MongoDB is more appropriate for small to medium non-critical sensor applications. Similar results were found by Jung et al. [33], with PostgreSQL surpassing MongoDB for handling structured data and MongoDB outperforming it when handling unstructured data.

Faraj et al. [34] examined how Oracle and MongoDB operate differently from relational and non-relational databases. While Oracle beats MongoDB in mathematical queries like aggregation functions, MongoDB dominates data retrieval performance for a big dataset in document-based mode.

Furthermore, Fatima et al. [35] and Rautnare et al. [36] compared, the performance of SQL (MySQL) and NoSQL (MongoDB) databases when managing large amounts of sensor data in the Internet of Things. MongoDB excels at many jobs, making it an excellent choice for handling IoT sensor data, especially given its adaptable, schema-less data architecture, whereas MySQL provides more consistent results.

However, Reetishwaree et al. [37] research offered a comparative examination of three database types: PostgreSQL (On-disk relational), HyperSQL (In-memory), and MongoDB (On-disk/In-memory). The research used sensor data from temperature, humidity, moisture, and water levels to assess how well these databases handle Insert, Select, Update, and Delete actions. According to the study, no single database performed better than all others in every situation. For example, NoSQL databases were excellent at updating and deleting data, whereas SQL databases were superior at performing select operations. The article emphasized the unique benefits and characteristics of NoSQL and SQL databases, arguing that they should be used in tandem rather than in opposition to one another.

The study conducted in Ref. [38] examined data models' function in Industry 4.0 (I4.0), specifically in Intelligent Manufacturing Systems (IMS). The study evaluated the suitability of relational (SQL) and non-relational (NoSQL) databases by comparing them and examining the five big data dimensions: volume, velocity, diversity, truthfulness, and value. To maximize performance while controlling complexity, the research highlighted that database selection can have a substantial impact on system performance.

PLC-based experiments have already happened. Shinkevich et al. [39] proposed a data collection system to optimize production resources in an enterprise. The system records data including temperature, weight, and pressure sensors on the physical condition of equipment using PLC controllers. The data is sent to the OPC platform for processing and analysis, along with historical data from the Manufacturing Execution System (MES) and PLC databases. The MES data is stored in a NoSQL database, HBase, due to the large and heterogeneous nature of the equipment parameters received from local PLCs, so the use of existing Relational Databases (RDB) is restricted.

Moreover, Covrig et al. [40] research focused on using web technologies under PLC control to simulate industrial processes. The study showed how more flexibility and customization may be obtained by replacing conventional SCADA systems with web-based interfaces like Node-RED and the S7Comm protocol. By achieving an average data transmission speed of 70 ms, the study effectively synchronized the simulation and PLC. Enhanced remote monitoring and control can be achieved by merging web technologies with industrial automation, as demonstrated by the data stored in a MySQL database through an Apache server.

The authors' research in Ref. [41] employed the MySQL database and the LSql library for communication to integrate databases with PLCs in an Industry 4.0 setting. In an experimental heating system, the research effectively recorded and stored data from a PLC, demonstrating the advantages of low entry costs and simple integration with pre-existing ICT infrastructure. Nevertheless, it encountered difficulties like excessive PLC memory utilization, synchronization problems, and cybersecurity issues.

However, the question of which database to utilize will always be there. The research implies that switching from relational to NoSQL databases may not be necessary. Organizations can make their choice depending on their unique demands since each type has advantages and disadvantages. For small datasets, relational databases are effective; for large data analytics, NoSQL is. Whether your application needs to handle Big Data beyond what an RDBMS can handle will determine whether you should use a NoSQL database. Compared to strict relational databases, NoSQL's flexible schema sometimes facilitates quicker development [24].

The decision to focus on this research was driven by the need to address critical gaps and challenges identified in the integration of industrial data systems. The review highlighted several key issues: the complexity of synchronizing data across different databases, the varying performance of relational and NoSQL databases in handling industrial data, and the persistent problem of data loss and delays in data transmission. Existing studies have shown the advantages and limitations of various databases, but a comprehensive examination of how these databases perform under real-time synchronization and data transfer conditions remains underexplored. This research seeks to fill this gap by evaluating how different databases manage synchronization and data integrity in an industrial automation context, addressing the specific problem of missing values and delays.

Furthermore, the literature revealed that while some solutions like Node-RED and various database systems have been used to improve industrial data handling, there is still a lack of in-depth analysis of their combined effectiveness in managing real-time data streams. By focusing on the integration of PLCs with Node-RED and multiple databases, this research aims to provide a clearer understanding of how to optimize data synchronization and minimize delays. The study's innovative approach, including the development of a predictive model for missing values, addresses the need for more reliable data management strategies in industrial automation. This focus aligns with the ongoing challenges in the field and aims to contribute practical solutions for enhancing data integrity and operational efficiency.

The key objectives of this study are to examine the fundamental behavior of integer data values being delivered to the databases and seek out any potential delays in this process. In particular, the effectiveness and dependability of data transfer systems will be examined, with particular attention paid to variables like latency, data transmission speed, and any issues that arise.

## 2 Materials and methods

The basic structure of the experiment is as follows: PLC is connected to the PC via an Ethernet cable. PC has 4 different databases. Databases are used in parallel to obtain the data of the counters all in the same timestamp. The databases under inspection are MySQL & Microsoft SQL (SQL), MongoDB, and Apache Cassandra (NoSQL).

A key component of this endeavor involves comparing how four different databases perform when it comes to storing and analyzing collected data. The target is to show the accuracy levels as well as processing speeds associated with each database platform inspected.

### 2.1 Software components

**Fig. 1:**
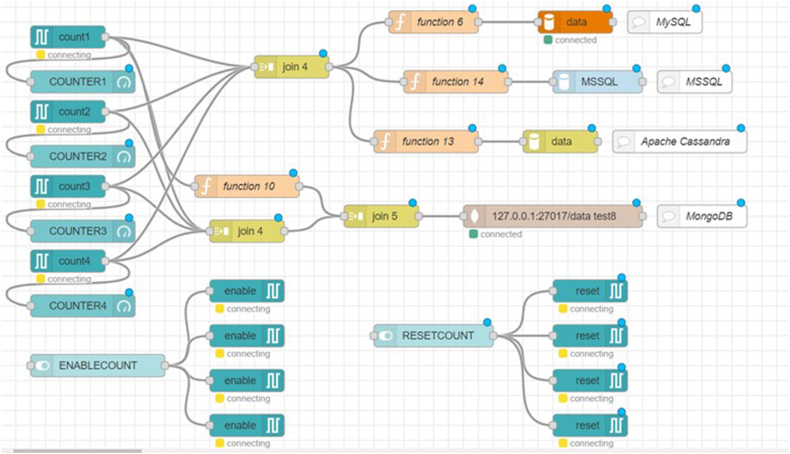
Flow of Node-RED to retrieve data from PLC S7-1200 and send them to SQL databases (MSSQL and MySQL) and NoSQL databases (MongoDB and Apache Cassandra).

PLC S7-1200 12/14 AC/DC/RLY was used to generate the data for the database. TIA V16 software was used to create the appropriate program, ladder diagram was used. The TC/IP connection has been used (ETHERNET Subnet), also PUT/GET communication from a remote partner is permitted, the system and clock memory were enabled taking into consideration the description of PLC S7-1200 that the clock memory is allowed up to 10 Hz as shown in Fig. 1.

The 4 databases are:

• MSSQL Database (Version 16.0.1000.6) also uses TCP/IP protocol and is controlled with SQL Server Management Studio (Version 16.0.1000.6) which is a SQL database.
• MySQL Database (version 8.0.34) also uses TCP/IP protocol for communication.
• MongoDB compass (version 1.37.0) is a graphical user interface tool used to interact with and manage MongoDB Server database (version 4.4) which is a NoSQL database.
• Apache Cassandra (version 3.11.4) uses the NoSQL Manager for Cassandra (version 6.4) as the graphical User Interface which is also a NoSQL database.

These versions were selected based on their relevance to current industrial applications and their compatibility with the study's objectives.

Node-RED (version 3.0.2) is used to communicate with the PLC, including node-red-contrib-s7 for accessing the S7 PLC, node-red-contrib-mssql for accessing the MSSQL database, node-red-dashboard, node-red-node-mongodb. Moreover, MATLAB (9.14.0.2286388 (R2023a) Update 3) was used to visualize the data and error detection. The communication flow is shown in Fig. 1.

It is critical to emphasize that MongoDB functions within the Node-RED platform in a manner distinct from those of the other three databases. Even though Apache Cassandra is classified as a NoSQL database, it has a unique data flow pattern such as the flow of the SQL databases. Examining Fig. 1 makes this difference clear, as the other databases require the utilization of a function that stores the counter and timestamp values for transmission. However, MongoDB, as a NoSQL database, only needs the timestamp from Node-RED as a function code; it does not need the counter numbers to be stored under a specific column or encoded using a function. In addition, MongoDB interface sets itself apart from the other three databases by not requiring the construction of a table.

### 2.2 Data generation and flow

In this research, a technique was developed for sending integer values utilizing TIA software version 16 and four counter networks operating at a clock frequency of 1 Hz [Hz] - that means one value every 1 s. The goal of constructing four counter networks is to always maintain consistent counter values by guaranteeing synchronization, which is the ability of all counters to operate simultaneously without one falling behind or operating more quickly than the others.

Moreover, a reference point is needed to compare the information obtained from the databases once the network connections have been established. By using the IP protocol of the TIA software, the technique entails generating a data log that records timestamps and counter values in the format (hh:mm:ss.ZZZ), where ZZZ stands for milliseconds. Milliseconds must be included to calculate the time interval between each collected counter value. This preliminary step is critical to the research since regulating the seconds alone would not provide us with precise timing for sending each counter value. It is important to consider the finer precision of time, especially when working with fractions of seconds, even if sending the counter data at 1-s intervals is suggested. This degree of accuracy guarantees that every counter value is precisely recorded and examined within the proper time range, enabling a more thorough understanding of the timing and synchronization of data transmission processes.

Moving to the Node-RED, according to the structure of each database, the data in the Node-RED platform is directed to four distinct function blocks. When the counter values reach the databases, time is supplied in the designated format (hh:mm:ss.ZZZ). While an SQL-based interface is used in Apache Cassandra, tables are created in MySQL and MSSQL to define each type of data. With MongoDB, all that must be constructed is the table name.

**Fig. 2:**
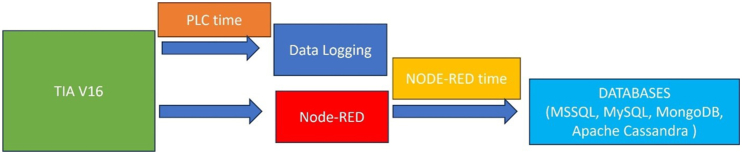
The Dataflow of the research.

Fig. 2, particularly highlights the saving of PLC time in the data log in addition to timestamps recorded in the databases, illustrates the straightforward methodology used in this study.

Initially after setting up, the PLC, the Node-RED, and the Four Databases, then tests were run to collect values in the range of 0–2000 counts. However, control could be made simpler from a single platform by utilizing the Node-RED platform to enable and reset the TIA software. The data from all four databases that includes the four counter values along with the timestamps was stored in xlsx format following each test, along with the reference data from the PLC's data log which also includes the four counter values along with PLC time, whenever the 2000 count mark was reached. Subsequently, ten tests were performed on different days and times and data from each test was gathered for comparison and analysis.

The first step was to synchronize the four databases. Accurate timestamps, including milliseconds, were demonstrated by Cassandra. Nevertheless, differences in the ways that data was kept led to disparities when comparing Cassandra timestamps with those of other databases. For example, MongoDB stored timestamps differently, so 14:55:16.6 would be 14:55:16.006, which can cause improper work in some cases. However, to solve the issue MongoDB timestamps were saved as JSON files and edited later.

There were similar differences between MySQL and MSSQL. Trigger time from the SQL databases was utilized to align timestamps with Node-RED time to fix those issues and guarantee synchrony across all databases. After the modifications, the timestamps appeared synchronously in all four databases.

As we mentioned in section 3.2, 2001 (with 0) integer values were created and sent to the databases from Node-RED with the clock-frequency 1 Hz. The first step of our examination was to confirm that every value had arrived at the databases. Analysis, however, showed that not all 2001 data were recorded in all 10 experiments. The difference in missing values was consistent across all databases and varied between tests. The PLC, Node-RED, and database connectivity issues were the cause of this disparity.

For each test, the total number of missing values, and the values that were not recorded are listed in Table 1. Additionally, every database showed the same missing values in every test. This assumes, that the reason for missing values does not depend on the databases, but the communication between databases and PLC, Node-RED.

**Table 1:** Missing values in all databases.

Tests	Missing Values	Number of Missing Values
Test 1	1,12,124,241,349,439,442,516,617,62,721,826,923, 926,1026,1119,1229,1317,1411,1501,1594, 1684,1693,1775,1879,1980	26
Test 2	1,70,76,173,177,269,271,369,462,552,652,748,842, 939,1035,1125,1222,1321,1414,1417,1511,1611, 1707,1810,1904	25
Test 3	83, 175, 267, 357, 444, 446, 539, 630, 721, 809, 898, 985, 1078, 1171, 1259, 1343, 1430, 1521, 1607, 1699, 1784, 1875, 1965	23
Test 4	86, 178, 267, 362, 446, 534, 624, 713, 799, 889, 977, 1078, 1176, 1267, 1365, 1465, 1560, 1662, 1763, 1766, 1861, 1962	22
Test 5	42, 146, 246, 355, 455, 566, 676, 779, 880, 982, 1089, 1199, 1201, 1321, 1430, 1555, 1673, 1776, 1878, 1978	20
Test 6	38, 42, 140, 144, 236, 241, 338, 343, 440, 444, 536, 542, 544, 638, 644, 743, 836, 840, 926, 932, 935, 1022, 1027, 1118, 1124, 1226, 1128, 1328, 1330, 1424, 1532, 1640, 1644, 1749, 1752, 1851, 1857, 1859, 1964	39
Test 7	49, 55, 58, 163, 169, 274, 276, 382, 384, 487, 489, 604, 709, 815, 920, 922, 1028, 1140, 1244, 1246, 1353, 1461, 1561, 1563, 1669, 1779, 1781, 1891	28
Test 8	26,32,34,127,225,298,397,400,493,496,596,680,686, 688,778,870,962,1050,1082,1171,1263,1354,1446, 1540,1635,1724,1814,1906.1997,1999	30
Test 9	31, 133, 233, 235, 335, 442, 545, 660, 770, 914, 1063, 1210, 1333, 1441, 1548, 1648, 1751, 1753, 1851, 1945	20
Test 10	85, 192, 292, 398, 501, 604, 708, 805, 807, 906, 1009, 1114, 1216, 1326, 1429, 1431, 1538, 1644, 1749, 1855, 1966	21

## 3 Results

There were created 10 tests under the above-mentioned, same conditions. After the comparison of these tests, we found that they produced similar results. According to this fact in the result section, Test 8, a random test, will present detailed information. Thirty missing values were identified in Test 8 in all four databases, so they have a total of 1971 values.

### 3.1 Node-RED normalized time to Node-RED synchronized time

**Fig. 3:**
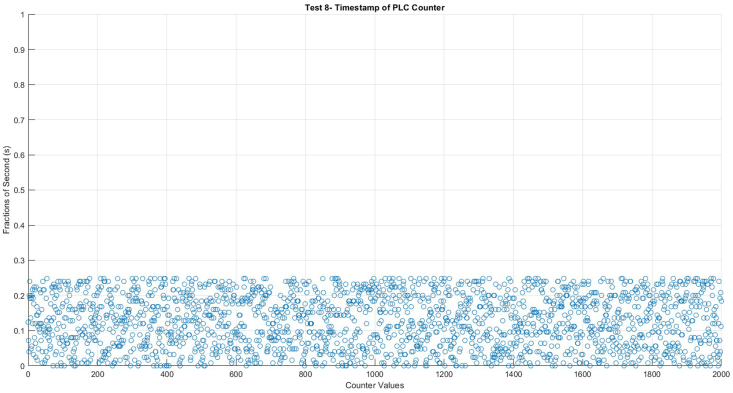
Timestamps of PLC counter.

As our initial step, Fig. 3 shows the PLC time extracted in milliseconds to ensure consistency in the counts. It shows that the counter values are sent every second between 0 and 0.248 ms. This visualization helps verify the consistency of count values over time and highlights any potential deviations and it justifies that no slips exist. On the other hand, the average time difference between the two values was 0.9995 s not 1 s exactly.

Moving to the four databases the calculated average time differences for each of them were 1.0146 s. This is determined by the Node-RED, not the different databases.

Determining the average transmitting time is essential since it offers a preliminary evaluation of the effectiveness of data transfer for all databases. The computed average transmitting time of 1.0146 s for the databases indicates a minor delay in the data transmission process when compared to the PLC's average time of 0.996 s. Through this analysis, it would help us to provide a deeper understanding related to the missing values. Table 2 contains the average time differences in the case of PLC, databases, the predicted missing values (equation (1)), and the real number of missing values.

(1) $$PMV=\left({NR}_{td}-\;{PLC}_{td}\right)∙n\text{,}$$

Where,

**Table 2:** Showing the results of equation (1) along with the Real number of missing values.

Tests	${\text{NR}}_{\mathrm{td}\;}$ (s)	${\text{PLC}}_{\mathrm{td}\;}$ (s)	n (s)	PMV	Real number of missing values
Test 1	1.0130	0.9999	1999.289	26.2	26
Test 2	1.0124	0.9996	2000.574	25.6	25
Test 3	1.0113	0.9995	2000.351	23.6	23
Test 4	1.0108	0.9994	2000.338	22.8	22
Test 5	1.0094	0.9995	1999.57	19.8	20
Test 6	1.0193	0.9996	1999.835	38.6	39
Test 7	1.0139	0.9995	2000.474	28.8	28
Test 8	1.0146	0.9995	1999.69	30.2	30
Test 9	1.0095	0.99945	1999.765	20.1	20
Test 10	1.0102	0.9995	2000.102	21.4	21

PMV is the predicted missing value,

${NR}_{td}$ is the average time difference of Node-RED in seconds,

${PLC}_{td}$ is the average time difference of PLC in seconds,

and n is the length of the test in seconds.

**Fig. 4:**
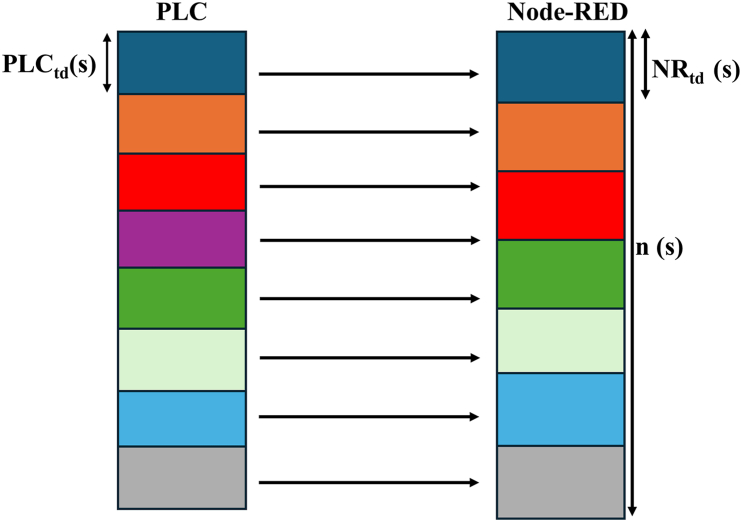
Systematic Illustration based on the predicted missing values (equation (1)).

To clarify the concept of predicted missing values as defined by (equation (1)), Fig. 4 illustrates the data transmission process from the PLC to Node-RED using color-coded data packets. Each color in the figure represents distinct counter values being transmitted.

The continuous sequence of colors demonstrates the normal flow of data packets, where each color corresponds to a specific packet sent at regular intervals. At a particular point in the timeline, however, one of the colors is missing (in this case, the purple packet). This interruption in the sequence signifies a missing value, indicating that a data packet was either lost or delayed during transmission. After this missing packet, the flow of colors resumes as usual, showing that, despite the temporary disruption, the data transmission process continues without further issues.

Fig. 4 highlights the practical application of the predictive equation by showing how inconsistencies in timing between Node-RED and the PLC can lead to missing values. By integrating the average time difference between these components, the equation estimates the number of missing values likely to occur. This visual representation highlights the impact of synchronization issues on data integrity and the occurrence of missing values in the data transmission process.

The relationship between the equation and the actual number of missing values is evident in Table 2. This alignment provides concrete proof of both the actual presence of missing values in the dataset and a delay in the data transmission process.

**Fig. 5:**
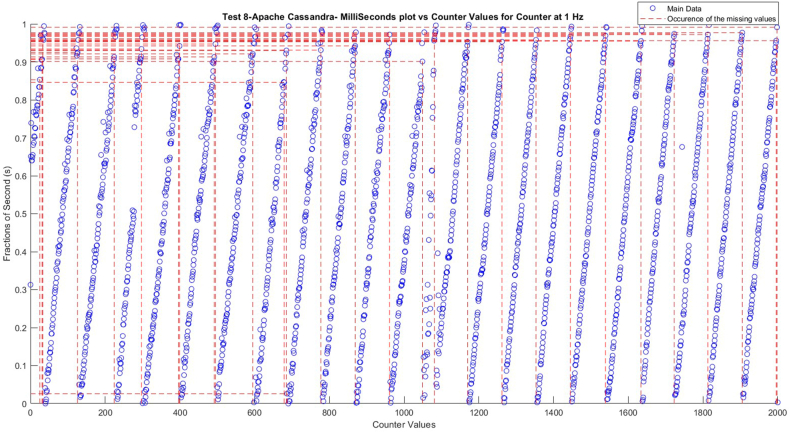
The occurrence of missing values according to the original Node-RED time.

Following Table 2, a plot was generated to visually depict the locations of missing values in Test 8, specifically focusing on Apache Cassandra as shown in Fig. 5. The plot shows that, with a few outliers at 0.853, 0.846, and 0.026 s, most missing values happened between 0.901 and 0.991 s. It is significant to observe that there are no values between 0.9 and the outliers (0.853, 0.846, and 0.026 s) because in these cases the time differences are high (1.913, 1.918, 1.946 s). The missing values appear after that counter number, which has specific values in milliseconds (in the case of Test 8 around 0.95). To understand the phenomenon more, time difference distribution was analyzed.

**Fig. 6:**
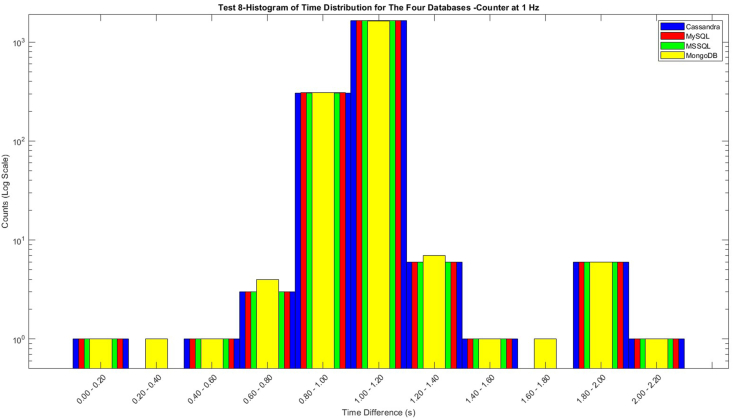
Histogram of the Time Difference between the four different databases.

Fig. 6, depicts a histogram of the time differences between the Node-RED sending time. It is essential to justify that the three databases were in sync completely. Some deviations occurred with MongoDB, recording count values at 0.20–0.40 and 1.60–1.80, but fewer at 1.20 these deviations occurred as was mentioned earlier regarding the different built form in Node-RED of the transmission of data from the PLC to the MongoDB interface. However, this does not happen many times (there is used logarithmic scale on axis Y).

Table 3 shows the different time values (original and normalized) and the missing values. When the PLC is sending the first value, it will be 0 s, the start of the test. It is important to note that there is a delay between PLC and Node-RED time, so Node-RED normalized time in this case just a rough approximation.

**Table 3:** Counter values, PLC time, Node-RED time, PLC normalized time, and Node-RED normalized time from values 0 to 29.

Counter Values	PLC time	Node-RED time	PLC normalized time	Node-RED normalized time
0	13:57:12.124	13:57:12.313	00:00:00.000	00:00:00.000
1	13:57:13.072	13:57:13.651	00:00:00.948	00:00:01.338
2	13:57:14.040	13:57:14.739	00:00:01.916	00:00:02.426
3	13:57:15.200	13:57:15.640	00:00:03.076	00:00:03.327
4	13:57:16.240	13:57:16.640	00:00:04.116	00:00:04.327
5	13:57:17.192	13:57:17.649	00:00:05.068	00:00:05.336
6	13:57:18.200	13:57:18.655	00:00:06.076	00:00:06.342
7	13:57:19.136	13:57:19.681	00:00:07.012	00:00:07.368
8	13:57:20.048	13:57:20.768	00:00:07.924	00:00:08.455
9	13:57:21.192	13:57:21.687	00:00:09.068	00:00:09.374
10	13:57:22.056	13:57:22.704	00:00:09.932	00:00:10.391
11	13:57:23.200	13:57:23.702	00:00:11.076	00:00:11.389
12	13:57:24.216	13:57:24.726	00:00:12.092	00:00:12.413
13	13:57:25.120	13:57:25.725	00:00:12.996	00:00:13.412
14	13:57:26.192	13:57:26.769	00:00:14.068	00:00:14.456
15	13:57:27.032	13:57:27.761	00:00:14.908	00:00:15.448
16	13:57:28.176	13:57:28.759	00:00:16.052	00:00:16.446
17	13:57:29.224	13:57:29.777	00:00:17.100	00:00:17.464
18	13:57:30.080	13:57:30.786	00:00:17.956	00:00:18.473
19	13:57:31.216	13:57:31.784	00:00:19.092	00:00:19.471
20	13:57:32.120	13:57:32.883	00:00:19.996	00:00:20.570
21	13:57:33.064	13:57:33.804	00:00:20.940	00:00:21.491
22	13:57:34.024	13:57:34.814	00:00:21.900	00:00:22.501
23	13:57:35.224	13:57:35.834	00:00:23.100	00:00:23.521
24	13:57:36.144	13:57:36.834	00:00:24.020	00:00:24.521
**25**	**13:57:37.168**	**13:57:37.853**	**00:00:25.044**	**00:00:25.540**
26	13:57:38.016	–	00:00:25.892	–
**27**	**13:57:39.144**	**13:57:38.970**	**00:00:27.020**	**00:00:26.657**
28	13:57:40.112	13:57:40.883	00:00:27.988	00:00:28.570
29	13:57:41.048	13:57:41.885	00:00:28.924	00:00:29.572

Also, Table 3 shows that the first missing value happens between numbers 25 and 27. The counter value is captured at counts 25 and 27. The two-capture time starts at 540 ms and finishes at 1657 ms, which is more than 1 s, and the PLC already changed the value to the next one (now 27). This is the main reason for the missing values.

**Fig. 7:**
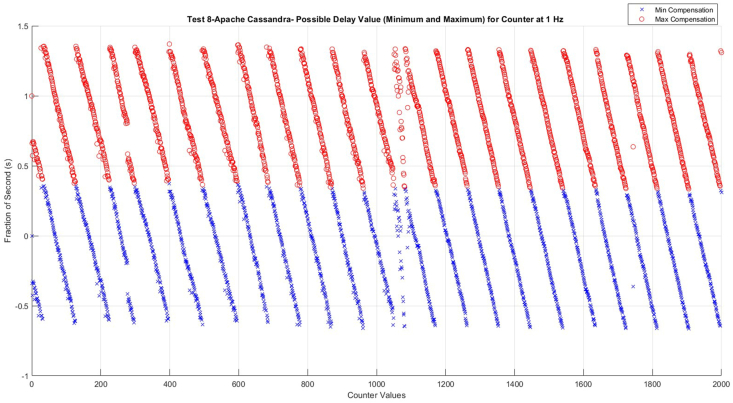
Possible delay (PD) values for test 8- Apache Cassandra.

The next task to get the real-time values is to determine the delay between the PLC and Node-RED. This delay could be caused by several things, such as network latency, PLC or Node-RED system processing delays, or inefficiencies in the data transfer protocol. Delay calculation is a complex task because any synchronization process has the same delay because of using the same network connection. On the other hand, it shows fluctuation as well (Fig. 7).

A new indirect method was developed to handle this problem. This expectation is based on the practical issue, that synchronization is possible in second level, with only millisecond synchronization needed. This also means that the delay is less than 1 s. The delay determination starts the calculation of the possible minimum and maximum compensation values (Table 4). As Table 4 shows, the Node-RED can get the same counter values from 000 ms to 999 ms. However, according to the rough normalized Node-RED time, when the missing values happen, the next value (now 27) shows a different pattern than the previous and the following values. The reason of it that the Node-RED sending time for count 27 had been recorded at 26.657 s, as it should ideally be between 27.000 and 27.999 s. This phenomenon helps to significantly limit the possibility of the delay values.

**Table 4:** Possible minimum and maximum compensation values.

Counter Values	Node-RED normalized time	Possible Minimum Compensation (PMINC)	Possible Maximum Compensation (PMAXC)
0	00:00:00.000	0.000	0.999
1	00:00:01.338	−0.338	0.661
2	00:00:02.426	−0.426	0.573
3	00:00:03.327	−0.327	0.672
4	00:00:04.327	−0.327	0.672
5	00:00:05.336	−0.336	0.663
6	00:00:06.342	−0.342	0.657
7	00:00:07.368	−0.368	0.631
8	00:00:08.455	−0.455	0.544
9	00:00:09.374	−0.374	0.625
10	00:00:10.391	−0.391	0.608
11	00:00:11.389	−0.389	0.610
12	00:00:12.413	−0.413	0.586
13	00:00:13.412	−0.412	0.587
14	00:00:14.456	−0.456	0.543
15	00:00:15.448	−0.448	0.551
16	00:00:16.446	−0.446	0.553
17	00:00:17.464	−0.464	0.535
18	00:00:18.473	−0.473	0.526
19	00:00:19.471	−0.471	0.528
20	00:00:20.570	−0.570	0.429
21	00:00:21.491	−0.491	0.508
22	00:00:22.501	−0.501	0.498
23	00:00:23.521	−0.521	0.478
24	00:00:24.521	−0.521	0.478
25	00:00:25.540	−0.540	0.459
**27**	**00:00:26.657**	**0.343**	**1.342**
28	00:00:28.570	−0.570	0.429
29	00:00:29.572	−0.572	0.427

Fig. 7 illustrates the possible minimum and maximum delay values in the case of every value. As it can see there is a clear border between the numbers. In this specific case, the border is around 0.35. To determine the exact delay value some additional calculation is done. Firstly, the values were separated into seven distinct ranges. For each range, the maximum of possible minimum compensation (*PMINC*) and the minimum of possible maximum compensation (*PMAXC*) compensation value is considered, to find the Average Delay ($A_{vg}D$) which is according to equation (2):

(2) $$A_{vg}D=\frac{\mathrm{MAX}\left(PMINC\right)+\mathrm{MIN}\left(PMAXC\right)}{2}$$

The $A_{vg}D$ shows at different intervals in Table 5. As can be seen, the delay has decreased continuously, which is the reason for the two average time differences (0.996 vs. 1.0146 s). If the PLC cycle time is faster than the Node-RED then it will change the value faster, which modifies the original delay time. The exact long-term behavior of the delay will be investigated in the future.

**Table 5:** The seven ranges with the minimum, maximum and Average Delay (A_vg_D).

Range	MAX (PMINC)	MIN (PMAXC)	Average Delay (A_vg_D)
0–300	0.3550	0.3760	0.3655
301–600	0.3650	0.3650	0.3650
601–900	0.3490	0.3490	0.3490
901–1200	0.3370	0.3400	0.3385
1201–1500	0.3310	0.3450	0.3380
1501–1800	0.3310	0.3380	0.3345
1801–2000	0.3220	0.3350	0.3285

**Fig. 8:**
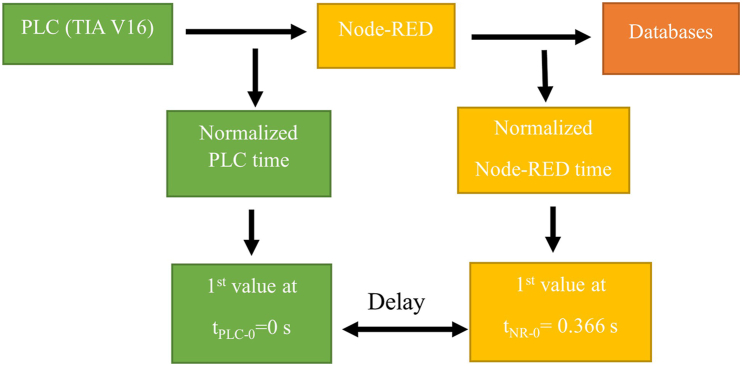
Post synchronized timestamps.

To determine the original $A_{vg}D$ and do the finalized Node-RED time can help to reach synchronized databases. Fig. 8 shows the structure of the real system, and Table 6 shows the values after synchronization. According to Table 6, it is seen, why 26 is the missing value and proves that the count value 27 is in synchronous with the Node-RED time as the other counter values.

**Table 6:** Synchronized time of Node-RED for the count values between 0 and 29.

Counter Values	PLC normalized time	Node-RED synchronized time
0	00:00:00.000	00:00:00.366
1	00:00:00.948	00:00:01.704
2	00:00:01.916	00:00:02.792
3	00:00:03.076	00:00:03.693
4	00:00:04.116	00:00:04.693
5	00:00:05.068	00:00:05.702
6	00:00:06.076	00:00:06.708
7	00:00:07.012	00:00:07.734
8	00:00:07.924	00:00:08.821
9	00:00:09.068	00:00:09.740
10	00:00:09.932	00:00:10.757
11	00:00:11.076	00:00:11.755
12	00:00:12.092	00:00:12.779
13	00:00:12.996	00:00:13.778
14	00:00:14.068	00:00:14.822
15	00:00:14.908	00:00:15.814
16	00:00:16.052	00:00:16.812
17	00:00:17.100	00:00:17.830
18	00:00:17.956	00:00:18.839
19	00:00:19.092	00:00:19.837
20	00:00:19.996	00:00:20.936
21	00:00:20.940	00:00:21.857
22	00:00:21.900	00:00:22.867
23	00:00:23.100	00:00:23.887
24	00:00:24.020	00:00:24.887
25	00:00:25.044	00:00:25.906
26	00:00:25.892	(missing value)
**27**	**00:00:27.020**	**00:00:27.023**
28	00:00:27.988	00:00:28.936
29	00:00:28.924	00:00:29.938

The key importance of the method is that it can create post-synchronization only according to the saved data. On the other hand, the synchronization works in millisecond-level accuracy. This method is independent of the physical network and devices. Also, synchronization can have a better chance to analyze the results. In the case of more equipment (more machines, more PLC) it will be more important to have post post-synchronization possibility.

### 3.2 Communication distribution

**Fig. 9:**
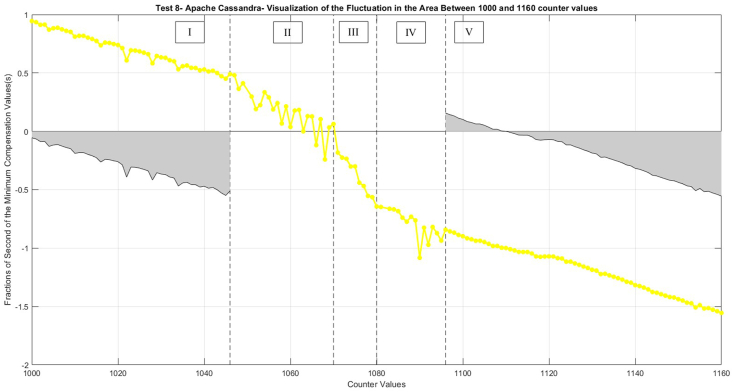
Visualization of the fluctuation area between 1000 and 1160 for Test 8- Apache Cassandra.

In the context of Test 8, the intermediary segment of the measurement, across the counts from 1000 to 1160, exhibits noteworthy fluctuations (Fig. 7). This range is vulnerable to external influences, thereby leading to variations in the Node-RED cycle time. Despite the functionality and reliability of the fundamental trends and models, a detectable increase in the Node-RED cycle time is evident (as depicted in Fig. 9). Within this region, inaccuracies become remarkable.

To see better the trends, compensation values were modified: increasing by +1 in the range between 1000 and 1049 (before the first missing value at 1050) and decreasing by −1 in the range between 1083 and 1160 (after the second missing value at 1082). Simultaneously, grayscale representations of the original minimum compensation values for these defined areas are plotted.

Fig. 9 is divided into five sections according to the performance. Sections I and V exhibit a normal slope, indicative of steady and consistent performance. In contrast, Section III displays a very high slope, representing a sharp increase in performance. Meanwhile, Sections II and IV are characterized by fluctuations, reflecting periods of instability and variability in the system's performance.

Section I indicates a noticeable decrease in slope, consistent with normal operating circumstances. The average time difference in this section is about 1.009 s, which is an acceptable time difference.

Moving on to Section II, which includes the count values of 1046 through 1070, Node-RED attempts to decrease delay by shortening the time difference between the consecutive counter values, but the fluctuations already started.

Section III exhibits a sudden and sharp drop in slope despite counts between 1071 and 1080 as it is a small region of counter values. In this instance, Node-RED struggles with time synchronization, leading to a significant temporal discrepancy of over 1.07 s between consecutive count values as it is considered a high time difference. This discrepancy, which indicates a significant departure from the average time difference, results in a reduced rate of communication speed, which in turn causes a significant 6.6 % decrease in speed.

Proceeding to Section IV, which includes counts 1081 through 1095, the Node-RED aims to correct temporal inconsistencies and return to the normal working conditions that chose fluctuation, like the condition in Section II (moderate communications speed decreasing).

In Section V, which consists of counts 1096 through 1160, Node-RED returns to its standard operating rhythm, replicating Section I's slope drop (as also shown in the grayscale that both areas have the same declining slope). Here, operational stability is restored, and the temporary fluctuation fades.

**Fig. 10:**
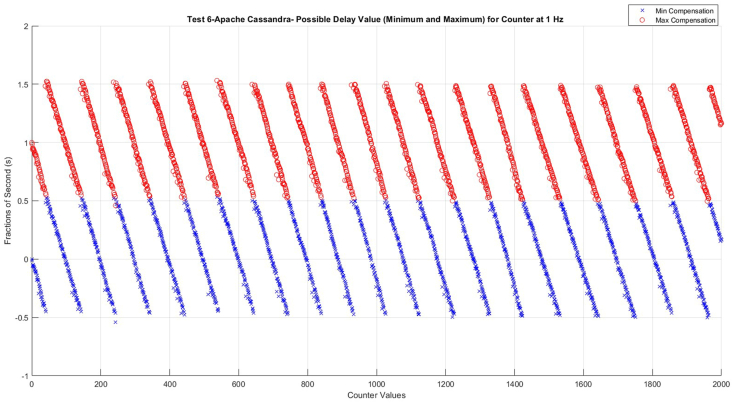
Possible delay (PD) values for test 6- Apache Cassandra.

Plots of the other nine tests, which present possible minimum and maximum compensation values, were also analyzed. However, they did not exhibit any fluctuations like those observed in Test 8 (Fig. 7). Nevertheless, Test 6 PD (Possible Delay) values (Fig. 10) are used as an example to demonstrate what the near-perfect working conditions of the possible minimum and maximum compensation values look like.

## 4 Conclusion

In an industrial environment is important to establish a reliable data collection process from the machines and sensors, through the network hardware and software components to the applied database. Nowadays the data collection is often done with such a high frequency, that the pipeline is not able to handle it without loss. We aimed to examine a typical artificial industrial data pipeline with PLC, regarding the problems associated with data loss occurring during data transmission and synchronization delays. Our analysis provided insights into optimizing these technologies to ensure reliable and efficient data management in evolving industrial environments.

A complex pipeline that connected the TIA software (Siemens PLC) to Node-RED and then to the four databases (MSSQL, MySQL, MongoDB, Apache Cassandra) was set up in the trial setting. The principal objective was to guarantee the accurate transmission of integer-based counter values to the databases at a precise clock rate of 1 Hz. The PLC data logging demonstrated extremely stable operation, accurately logging every counter value between 0 and 2000 at a speed of 0.9999 on average. The faultless functionality of the PLC is highlighted by this excellent performance.

Furthermore, an intriguing observation emerged as all four different databases exhibited synchronous behavior, displaying identical counter values along with their corresponding timestamps. This synchronicity indicated that despite the presence of both SQL and NoSQL databases in our study, they functioned harmoniously in sync. However, during this synchrony, a notable phenomenon unfolded; the occurrence of the same missing values in all four databases throughout the tests; remarkably the missing values varied across the ten tests.

The phenomenon of missing values delineates the absence of certain counter values along their timestamps, despite the systematic operation of the PLC and data transmission processes. This occurrence underscores the intricacies of data capture and highlights the need for comprehensive analysis to understand the underlying factors contributing to the absence of these values.

The key innovative contributions of this research are now evident. Firstly, it introduces a novel predictive model for estimating missing values during data transfer, which significantly enhances the understanding and management of synchronization issues. Secondly, the study demonstrates how integrating Node-RED with various databases (MSSQL, MySQL, MongoDB, and Apache Cassandra) offers a unique approach to optimizing data synchronization and minimizing delays. These contributions provide practical solutions for improving real-time data collection and processing efficiency in industrial settings, filling a critical gap in existing methods and advancing the field.

Based on the experiments and their corresponding results, a predictive formula was developed to anticipate the number of missing values (PMV) occurring during data transfer. The equation yielded an approximation of the missing values that closely reflected the actual results. This predictive model assumes a critical role in preemptively estimating missing values, thereby facilitating a smoother understanding of their occurrence within large-scale data transfer processes.

Additionally, the research underscored the critical role played by PLC time and Node-RED normalized time in identifying delays and determining minimum and maximum compensation values. Based on the new model, accurate synchronized Node-RED time was achieved. This predictive capability not only enhanced the understanding of the underlying mechanisms governing counter synchronization but also provided practical insights for optimizing synchronization protocols in real-world applications.

Deeper insights into the findings from Test 8 reveal the dynamic nature of the system, as evidenced by significant fluctuations observed in the Node-RED cycle time within the intermediary segment of the measurement. This addresses operational challenges that may arise in industrial automation settings.

The implications of our findings for industrial data systems are substantial. While previous research emphasized NoSQL databases' advantages in managing massive amounts of data, our findings demonstrate that missing values and synchronization problems are systemic problems that impact both SQL and NoSQL databases. This indicates that data integrity challenges are more likely related to the integration methods used rather than the specific types of databases. By comparing our findings with existing studies, we provide a deeper understanding of the limitations of current data management approaches and propose improvements. Our research aims to enhance operational performance and data collection efficiency through better synchronization techniques and predictive models.

As a result, this research provides valuable background insights for optimizing data transmission and synchronization in large-scale industrial settings in real-life applications. By developing predictive models and refining synchronization mechanisms, real-life automation can enhance data collection efficiency, improve operational performance, and make more informed decisions. These findings offer practical applications for optimizing data management processes and enhancing overall productivity in big industrial automation.

In future work, exploration of the integration of different types of data transmission beyond integer values and integration of different clocks to enhance synchronization accuracy will be pursued. Investigation of the long-term behavior of delay will also be a focus of future research endeavors. These efforts aim to deepen our understanding of counter-synchronization dynamics and pave the way for more robust and efficient industrial automation systems.

## Funding

Supported by the project No. 2019–1.3.1-KK-2019-00011 financed by the 10.13039/501100012550National Research, Development and Innovation Fund of Hungary under the Establishment of Competence Centers, Development of Research Infrastructure Program funding scheme.

## Data availability statement

Data will be made available on request.

## CRediT authorship contribution statement

**Ayah Hijazi:** Writing – original draft, Visualization, Methodology, Formal analysis, Conceptualization. **Mátyás Andó:** Writing – review & editing, Supervision, Project administration, Methodology, Conceptualization. **Zoltán Pödör:** Writing – review & editing, Supervision, Project administration.

## Declaration of generative AI and AI-assisted technologies in the writing process

During the preparation of this work, the authors used ChatGPT and QuillBot AI in order to improve only readability and language. After using this tool, the authors reviewed and edited the content as needed and take full responsibility for the content of the publication.

## Declaration of competing interest

The authors declare that they have no known competing financial interests or personal relationships that could have appeared to influence the work reported in this paper.

## Appendix

This Appendix provides detailed descriptions and code snippets for key functions used in our Node-RED workflows to manage and process industrial data. These functions are crucial for tasks such as generating timestamps, formatting data, and building SQL and MongoDB queries, which allow for effective data insertion to the four databases. Every function has been designed to work with parts of our system to maintain data integrity and facilitate real-time processing.

### Function 6: MySQL Data Insertion with Timestamp

This function constructs an SQL INSERT statement to store the four counter values along with the timestamp in the MySQL database. The timestamp is formatted as YYYY-MM-DD hh:mm:ss.ZZZ. and it constructs an SQL INSERT statement to store the data in a table named test8.

### Function 14: MSSQL Data Insertion with Timestamp (MSSQL)

This function formats the four counter values and inserts them into a Microsoft SQL Server database. It creates a timestamp in the YYYY-MM-DD hh:mm:ss.ZZZ format and construct an SQL INSERT statement to store the data in the dbo.test8 table.

### Function 13: SQL Data Insertion with Timestamp (Apache Cassandra)

Function 13 is used for inserting data into an Apache Cassandra database. Although Cassandra is a NoSQL database, its interface in our setup uses the NoSQL Manager for Cassandra, which allows for SQL-like queries. As a result, the code used for data insertion closely resembles that used for MySQL databases.

### Function 10: MongoDB Data Preparation

Function 10 formats the current timestamp and prepares a message for insertion into a MongoDB collection. It sets the collection name and constructs a payload with the timestamp to be stored in the test8 collection.

Moreover, the table or collection name (test8 in this example) can be dynamically formatted based on the specific test being run, allowing the data to be saved in the appropriate location depending on each test's requirements.
